# A Hydroethanolic Leaf Extract of *Persicaria lanigera* Possesses Antinociceptive Activity through Cytokine and Glutamatergic Pathways *In Vivo*

**DOI:** 10.1155/2021/5586789

**Published:** 2021-06-18

**Authors:** Ernest Obese, Elvis O. Ameyaw, Robert P. Biney, Isaac T. Henneh, Nora Jackson, Daniel Anokwah, Augustine Brah, Esther E. Oppong, Emmanuel A. Adakudugu

**Affiliations:** ^1^School of Pharmacy and Pharmaceutical Sciences, College of Health and Allied Sciences, University of Cape Coast, Cape Coast, Ghana; ^2^Department of Pharmacognosy, Faculty of Pharmacy and Pharmaceutical Sciences, Kwame Nkrumah University of Science and Technology, Kumasi, Ghana; ^3^Department of Biomedical Sciences, School of Allied Health Sciences, College of Health and Allied Sciences, University of Cape Coast, Cape Coast, Ghana

## Abstract

*Persicaria lanigera* is used traditionally to treat pain. The antinociceptive properties of the hydroethanolic leaf extract of *Persicaria lanigera* (PLE) were evaluated in rats and mice. Mice were pretreated orally with PLE (30, 100, and 300 mg kg^−1^) and evaluated for antinociceptive effects in the acetic acid-, glutamate-, and formalin-induced nociception models. Additionally, mechanical hyperalgesia models were used to evaluate PLE's influence on TNF-*α*- and IL-1*β*-induced hyperalgesia in rats. In the acetic acid-induced nociception model, 100 mg kg^−1^ PLE exhibited the highest antinociceptive activity of 95.13 ± 9.52% at *p* < 0.0001, followed by the 300 mg kg^−1^ (85.44 ± 5.75%; *p* < 0.0001) and then the 30 mg kg^−1^ (67.95 ± 18.55%; *p* < 0.01), compared to morphine 3 mg kg^−1^ i.p. (86.97 ± 9.52; *p* < 0.0001). PLE (30, 100, and 300 mg kg^−1^) also showed significant (*p* < 0.05) antinociceptive effect in phase two of the formalin-induced nociception with % inhibitions of 66.88 ± 12.17, 75.12 ± 9.01, and 89.12 ± 4.32%, respectively, compared to 3 mg/kg morphine (97.09 ± 2.84%). Similarly, PLE (30, 100, and 300 mg kg^−1^) significantly reduced pain in the glutamate-induced nociception model with % inhibitions of 79.28 ± 8.17, 90.54 ± 5.64, and 96.49 ± 1.43%, respectively, whereas ketamine (5 mg/kg i.p.) reduced nociception to be 59.94 ± 18.14%. All doses of PLE significantly reduced nociceptive scores in TNF-*α*- and IL-1*β*-induced mechanical hyperalgesia (*p* < 0.01). Similarly, PLE significantly inhibited bradykinin-induced nociception. The hydroethanolic extract of *Persicaria lanigera* has antinociceptive effects; this is the first scientific report providing evidence to validate its traditional use for the management of pain.

## 1. Introduction

Nociception refers to the neural processes of encoding and processing noxious stimuli, which results from the stimulation of sensory fibres by noxious stimuli. Pain is often the main symptom in the clinical diagnosis of various human and animal diseases [1]. As a component of the human immune and inflammatory response system, pain is experienced when sensory signals are transmitted to the nervous system to cause a motor reaction toward harmful stimuli [2]. While it is a defensive immune response to injury, it adversely affects the quality of human life by reducing productivity, cognitive capacity and also induces anxiety, depression, and emotional instability [3]. Proinflammatory cytokines, as well as inflammatory mediators such as bradykinins and prostaglandins, play a crucial role in sensitizing nociceptors directly or indirectly through the trigger of various molecular mechanisms that lead to hyperalgesia or hypernociception [4]. The administration of TNF-*α* and interleukins IL-1*β*, IL-8, IL-12, IL-15, and IL-18 in rodents via intradermal route has been shown to produce a heightened and prolonged mechanical sensitization and hypernociception [5].

Pain remains a major cause of morbidity and mortality worldwide and the current drugs used to treat pain cannot fully relieve pain due to inadequate effectiveness, especially in chronic pain [6]. Moreover, the use of opioid analgesics, NSAIDs, and corticosteroids in pain management normally present moderate-to-severe adverse effects and drug dependence [1, 6]. Hence, the need for new analgesics is essential. As such, it is imperative to develop safe, effective, and potent analgesics, particularly for chronic pain. Currently, attention has been shifted to natural product medicines in most countries because they are readily available and less expensive and presumably have fewer side effects [7]; hence, they are more acceptable to the majority of folks. Plant derivatives continue to play a major role in the drug discovery process and also serve as a vital source of natural products with potential analgesic properties and hence could be promising agents for the development of safe and efficacious analgesics [6]. One of such promising plants is *Persicaria lanigera* (R.Br.) Soják, a member of the genus *Persicaria* belonging to the family Polygonaceae, is generally referred to as smartweeds. Numerous medicinal applications have been attributed to the Polygonaceae family, such as the treatment of ulcerative colitis, intestinal parasites, asthma, bronchitis, inflammatory diseases, and diarrhea [8]. The *Persicaria* species have been reported to possess various medicinal properties and are also rich in phytochemical constituents such as flavonoids, anthraquinones, and terpenoids. They have been used traditionally as agents for the treatment of colic pain, knee pain, rheumatic pain, skin pain, menstrual pain, and others [8, 9]. Significant antinociceptive activity in some nociceptive models in mice has been previously demonstrated by a methanolic extract of the leaves of *Persicaria hydropiper* [9]. Furthermore, *Persicaria chinensis* also exhibits anti-inflammatory and hepatoprotective properties in lipopolysaccharide- (LPS-) induced hepatitis in mice [10]. This suggests that *Persicaria lanigera* may also have analgesic properties. This is further strengthened by the fact that a decoction of *Persicaria lanigera* leaves has been used traditionally for managing pain and some inflammatory conditions in some communities in Ghana. Therefore, the lack of scientific data authenticating its antinociceptive activity in any scientific model despite being traditionally used inspired us to investigate the antinociceptive effect of hydroethanolic leaf extract of *Persicaria lanigera* in both peripheral and central pain models in mice and rats.

## 2. Materials and Methods

### 2.1. Drugs and Chemicals

The following chemicals and drugs were used in the study: morphine hydrochloride was obtained from Phyto-Riker, Accra, Ghana. Interleukin-1*β*, TNF-*α*, L-glutamic acid, and bradykinin acetate were obtained from Sigma-Aldrich Inc., St. Louis, MO, USA. Ketamine HCl from Brotex Medica, Trittau, Germany, and captopril from Teva UK Ltd. Acetic acid and formalin were also purchased from the British Drug House, Poole, England.

### 2.2. Plant Collection, Identification, and Extraction

Fresh *Persicaria lanigera* leaves were collected from Effutu (5°12′0″ N, 1°19′0″ W), off the Jukwa road, Cape Coast, Ghana, in November 2018. The plant was identified and authenticated by Mr. Fynn at the School of Biological Sciences Herbarium Unit, University of Cape Coast, Ghana. The plant material collected was transported in a clean dry bag and the leaves of *Persicaria lanigera* were air-dried at room temperature for 21 days. Leaves weighing (2 kg) were pulverized into a fine powder with the aid of a hammer mill at the Department of Chemistry, University of Cape Coast. To obtain the extract, 500 g of the powder was soaked in 2.5 L of 70% *v*/*v* ethanol for three days (72 h). The extract was filtered using a Buchner funnel and Whatman's filter paper No.1, and the filtrate was concentrated at 50°C under reduced pressure and vacuum using a rotary evaporator. The extract of *Persicaria lanigera* leaves (PLE) was then dried in a desiccator.

### 2.3. Animals

Male ICR (Institute of Cancer Research) mice (20–25 g) and Sprague Dawley rats (250–300 g) of both sexes were purchased from Noguchi Memorial Institute for Medical Research, University of Ghana, Legon, Ghana, and housed in the animal facility of the Department of Biomedical Sciences, University of Cape Coast (UCC). They were kept in plastic cages (37 cm × 47 cm × 18 cm) with softwood shavings (sawdust) as beddings. The animals were maintained under laboratory conditions of 23 ± 2°C temperature and humidity-controlled room (40–45%) at alternate 12 h light/dark cycle. They were fed with a normal commercial pellet diet and given clean water *ad libitum.* The animals were acclimatized to the laboratory environment for seven (7) days before performing the experiments. All procedures and techniques used in these studies were performed following the National Institute of Health Guidelines for the Care and Use of Laboratory Animals. All protocols used were approved by the Animal Ethics Committee of the University of Cape Coast with the identifier UCCIRB/CoHAS/2018/116.

### 2.4. High-Performance Liquid Chromatography (HPLC) Fingerprint

The PLE (200 mg) was reconstituted in 10 mL of 70% hydroethanol to obtain a stock solution (2% *w*/*v*). The solution (about 2 mL) was filtered using a 0.45 *μ*m membrane filter into the HPLC sample vial. The filtrate was, then, analysed using a PerkinElmer Flexar HPLC system with an autosampler, binary pump, Photodiode Array detector, and an online degasser. The elution was done on the Zorbax 300SB-C18 analytical column (4.6 × 250 mm, 5 *μ*m) from Agilent Technologies with an injection volume of 20 *μ*L and an acquisition wavelength of 280 nm. The solvent system of 0.05% trifluoroacetic acid (A) and methanol (B) was used as the mobile phase through a gradient elution method at a flow rate of 1 mL/min (at 26°C). The elution was programmed as follows: 0–3 min, 15% B; 3–23 min, 90% B; 23–26 min, 90% B; 26–27 min, 15% B; 27–32 min, 15% B [11].

### 2.5. Acetic Acid-Induced Writhing Test

The test was carried out as described by [12]. All drugs were freshly prepared. PLE (30, 100, and 300 mg kg^−1^) or morphine (3 mg kg^−1^, i.p.) were administered to the mice after they had received intraperitoneal injection of acetic acid (0.2 mL kg^−1^ of 0.4% *v*/*v*) 1 h (p.o.) or 30 min (i.p.) earlier. The mice were then individually placed in testing chambers (Perspex chamber of 15 cm × 15 cm × 15 cm). Injection of the acetic acid induced a nociceptive behaviour which was expressed as an exaggerated extension of the abdomen combined with the outstretching of the hind limbs (writhing). Responses were captured on a camcorder for 30 min for further analysis, starting 5 min after acetic acid administration. Tracking of the animals was done using a public domain software JWatcher™, Version 1.0 (University of California, LA, USA, and Macquarie University, Sidney, Australia), to assess the frequency and duration of writhing each 5 min for the entire 30 min. The total number of writhes per every 5 min was determined, which was used to plot time-course curves. An AUC graph was plotted out of the time-course curve, indicating the total nociceptive score. The percentage of inhibition was calculated as follows:

(1) $$\begin{array}{c} \%\text{ inhibition}=\frac{C-T}{C}\times 100, \end{array}$$

where *C* is the vehicle control group score for each phase and *T* is the treated group score value for each phase.

### 2.6. Formalin-Induced Nociception

In this model, formalin was used to induce nociception in accordance with [13]. Animals (ICR mice) were placed in five groups (*n* = 5). Nociception was induced by administering 10 *μ*L of 5% *v*/*v* formalin into the right hind paws of mice to induce nociceptive behaviour. Paw licking was measured from 0 to 5 min and 15 to 60 min after formalin treatment for the neurogenic and inflammatory stages, respectively. The mice were orally given PLE (30, 100, and 300 mg kg^−1^) 1 h before induction or morphine (5 mg kg^−1^, i.p.) 30 min before induction. The nociceptive scores were similarly measured as already shown in the acetic acid model in Section 2.5. The results for the time-course curve and the areas under the curve for each treatment were determined and plotted.

### 2.7. Glutamate-Induced Nociception

The glutamate-induced nociception model was carried out [14] with some modifications. The mice were placed in five groups (*n* = 5). To induce nociceptive effect, glutamate (20 *μ*L, 10 *μ*mol/paw) was administered into the ventral surface of the right hind paw of mice after they had been pretreated for 1 h with PLE (30, 100, and 300 mg kg^−1^) or 30 min after ketamine (5 mg kg^−1^, i.p.). Animals in the control group were given normal saline (10 mL kg^−1^, p.o.). Animals were observed for 15 min. The nociceptive scores were similar, as already shown in the acetic acid model described above. The total number of licks per every 5 min time block was obtained and was used to plot the time-course curve from which the areas under the curve (AUCs) were calculated.

### 2.8. Tumour Necrosis Factor-Alpha- (TNF- *α*-) Induced Hyperalgesia

TNF-*α* was used to induce mechanical hyperalgesia in rats as described elsewhere [15] with modifications [4]. Five groups of rats (*n* = 5) were pretreated with vehicle (10 mL kg^−1^, p.o.) and PLE (30, 100, and 300 mg kg^−1^) for 1 h or morphine (3 mg kg^−1^, i.p.) for 30 min. This was followed by intraplantar injection of 20 *μ*L of TNF-*α* (2.5 pg/paw) into the right hind paw. Mechanical nociceptive thresholds of the rats were measured in their paws hourly for 5 h after TNF-*α* injection by using an algesimeter (Model No. 15776, Ugo Basile, Comerio, Varese, Italy) [16]. Paw withdrawal thresholds (PWTs) were assessed as the pressure (grams) required to elicit paw withdrawal with a cutoff point of 250 g. Change in the hyperalgesic state was calculated as a percentage of the maximum possible effect (% MPE) according to the following formula:

(2) $$\begin{array}{c} \%\text{ MPE}=100\times \frac{\left(\text{PWT}-\text{CT}\right)}{\left(250\text{ g}-\text{CT}\right)}, \end{array}$$

where PWT is the paw withdrawal threshold and CT is the control (baseline) threshold.

### 2.9. Interleukin-1 Beta*-* (IL-1*β-*) Induced Hypernociception

Mechanical hypernociception was induced with IL-1*β* in rats as described by [15] with slight modifications [4]. Five groups of rats (*n* = 5) received pretreatment with either vehicle (10 mL kg^−1^, p.o.), PLE (30, 100, and 300 mg kg^−1^) for 1 h, or morphine (3 mg kg^−1^, i.p.) for 30 min before intraplantar injection of IL-1*β* (20 *μ*L, 1 pg/paw) into the right hind paw. Mechanical nociceptive thresholds were measured as described above in Section 2.7.

### 2.10. Bradykinin-Induced Hyperalgesia

The hyperalgesia induced in all the animals treated with bradykinin was similar to that described by [17]. The baseline measurements were performed, and five groups of rats were pretreated with the extract (30, 100, and 300 mg kg^−1^), morphine (3 mg kg^−1^), or vehicle after the baseline measurements and one hour later received an intraplantar injection of bradykinin (20 *μ*L: 10 nmol/paw). Animals were pretreated with captopril, 5 mg kg^−1^ s.c. (an angiotensin-converting enzyme inhibitor) 1 h before intraplantar injection of bradykinin to prevent bradykinin degradation. After intraplantar injection of bradykinin, the paw withdrawal thresholds (PWTs) were measured at 1, 2, 3, 4, and 5 h after bradykinin. Mechanical nociceptive thresholds were measured as described above in Section 2.7.

### 2.11. Statistical Analysis

All the data obtained were presented as the mean ± standard error of mean (SEM). The time-course curves in all the results in the study were analysed using a two-way analysis of variance (ANOVA) with Bonferroni's post hoc test followed by Bonferroni's multiple comparison tests. One-way ANOVA with either Newman–Keuls *post hoc* tests were used to determine differences between treatment groups (areas under the curves). GraphPad Prism version 7.0 (GraphPad Prism Software, San Diego, CA) for Windows was used to perform all statistical analyses, with *p* < 0.05 considered statistically significant for all tests.

## 3. Results

### 3.1. Plant Extraction

The yield obtained from the extraction was 3.8% *w*/*w*.

### 3.2. HPLC Fingerprint

**Figure 1:**
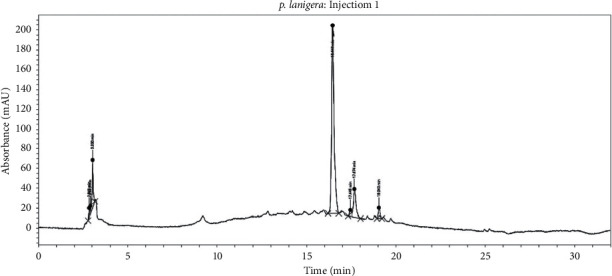
HPLC chromatogram of PLE.

The result of the HPLC analysis showed seven prominent peaks in the chromatogram (Figure 1), which represent various constituents in the PLE. The peaks were identified with their retention time, peak area, and height (Table 1).

### 3.3. Effect of PLE on Acetic Acid-Induced Writhing

**Figure 2:**
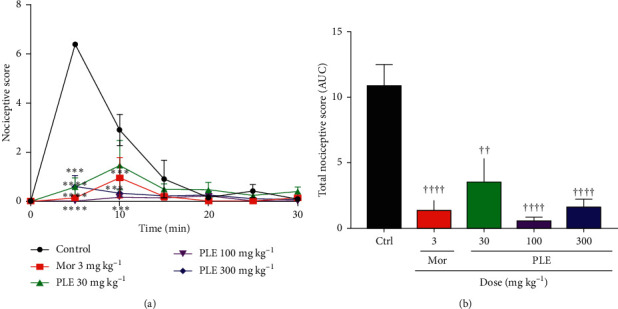
The effect of PLE on (a) nociceptive score and (b) total nociceptive scores in acetic acid-induced writhing in mice. Data are presented as mean ± SEM. The symbols ^∗^ and † indicate the significance levels compared to respective controls: ^*∗∗∗∗*^*p* < 0.0001, ^*∗∗∗*^*p* < 0.001 (two-way ANOVA followed by Bonferroni's post hoc test); ^††††^*p* < 0.0001, ^††^*p* < 0.01 (one-way ANOVA followed by Newman–Keuls post hoc test).

From Figures 2(a) and 2(b), there was a significant reduction in the total nociceptive score by the extract (30, 100, and 300 mg kg^−1^) and morphine (3 mg kg^−1^). The time-course curve (Figure 2(a)) revealed that PLE gave a significant reduction in the total nociception from 0 to 10 min. At 5 min, the extract (30, 100, and 300 mg kg^−1^) significantly (*p* < 0.0001) reduced the total nociception. However, at 10 min, 100 mg kg^−1^ and 300 mg kg^−1^ significantly reduced the total nociception (*p* < 0.0001), while 30 mg kg^−1^ significantly reduced the total nociception to a lesser degree (*p* < 0.05). The calculated AUC (Figure 2(b)) revealed that the extract PLE of 100 mg kg^−1^ and 300 mg kg^−1^ significantly reduced the total nociception (*p* < 0.0001) followed by PLE 30 mg kg^−1^ (*p* < 0.001). The % inhibitions of nociception were calculated using the AUCs and the results obtained were 67.95 ± 18.55, 95.13 ± 2.75, and 85.44 ± 5.75 respectively, for 30, 100, and 300 mg/kg of PLE. Correspondingly, from the time-course graph, morphine (3 mg kg^−1^) also significantly reduced the total nociception induced by acetic acid at 5 min (*p* < 0.0001) and 10 min (*p* < 0.001). The calculated AUC (Figure 2(b)) also revealed significant (*p* < 0.0001) inhibition of writhing of 86.97 ± 9.52.

### 3.4. Effect of PLE on Formalin-Induced Paw Licking

**Figure 3:**
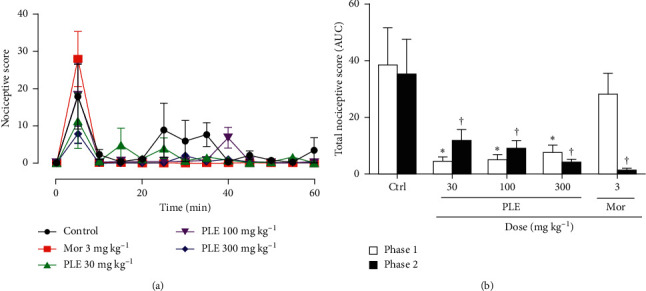
The effect of PLE on formalin-induced nociception: (a) time-course curve (TCC) for the antinociceptive activity of PLE; (b) AUC of the time-course curve. Results are presented as means ± SEM of the latency period (*n* = 5). ^*∗*^*p* < 0.05 and ^†^*p* < 0.05 compared to respective controls (one-way ANOVA followed by Newman–Keuls' post hoc test).

For the formalin-induced nociception model used, phase 1 (Figure 3(a)), the scores for PLE at doses of 30, 100, and 300 mg kg^−1^ were significantly smaller (*p* < 0.05) than those in the control group. Although morphine at a dose of 3 mg kg^−1^, i.p., had an analgesic effect, the effect observed was not significant compared to the negative control as expected in theory. In the second phase (10–60 min), PLE (30, 100, and 300 mg kg^−1^) showed a significant (*p* < 0.05) antinociceptive effect in phase two of the formalin-induced-nociception with % inhibitions of 66.88 ± 12.17, 75.12 ± 9.01, and 89.12 ± 4.32%, respectively, and % inhibition of 3 mg/kg morphine was 97.09 ± 2.84% (*p* < 0.01). The nociceptive scores for PLE (30, 100, and 300 mg kg^−1^) and morphine (3 mg kg^−1^, i.p.) were all significantly lower than that of the control group (*p* < 0.05 and *p* < 0.01, respectively) (Figure 3(b)).

### 3.5. Effect of PLE on Glutamate-Induced Nociception

**Figure 4:**
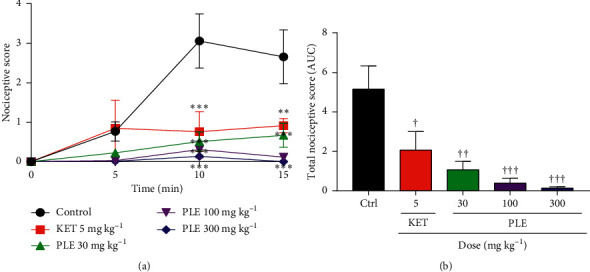
Effect of PLE (30, 100, and 300 mg kg^−1^) and ketamine (5 mg kg^−1^, i.p.) on glutamate-induced nociceptive pain: (a) the time-course curve; (b) AUC of the time-course curve. Each data represents the mean of 5 animals and the error bars indicate SEM. ^∗^ and † indicate the significance levels compared to respective controls: ^*∗∗∗*^*p* < 0.001 (two-way ANOVA followed by Bonferroni's post hoc test); ^†††^*p* < 0.001, ^††^*p* < 0.01, ^†^*p* < 0.05 (one-way ANOVA followed by Newman–Keuls post hoc test).

Administration of PLE (30, 100, and 300 mg kg^−1^) and ketamine (5 mg kg^−1^, i.p.) produced significant reductions in paw licking behaviour induced by glutamate during the 15 min observational period (Figure 4(a)). The total nociceptive effect of glutamate was significantly reduced by both PLE and ketamine (Figure 4(b)). All doses of PLE significantly increased antinociception in a dose-dependent manner compared to control (saline). The overall results depict that all the doses of the extract and control (ketamine) showed antinociceptive activity compared to the vehicle or the negative control group. The total nociceptive score (AUC) graph (Figure 4(b)) reveals that treatment with PLE (30, 100, and 300 mg kg^−1^) significantly reduced pain in the glutamate model with % inhibitions of 79.28 ± 8.17, 90.54 ± 5.64, and 96.49 ± 1.43%, respectively, whereas ketamine reduced nociception by 59.94 ± 18.14%. The extract produced a dose-dependent inhibition at *p* < 0.001 for the 100 mg kg^−1^ and 300 mg kg^−1^ doses and at *p* < 0.01 for the 30 mg kg^−1^. Additionally, the induced nociceptive behaviour was greatly reduced in the extract-treatment animals compared to that in ketamine-treated animals (*p* < 0.05).

### 3.6. Effect of PLE on TNF-*α*-Induced Hyperalgesia

**Figure 5:**
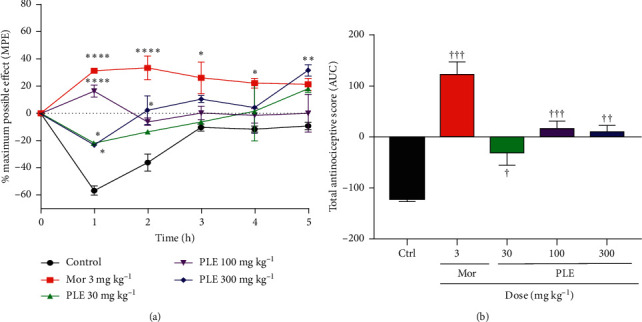
The effect of PLE and morphine on TNF-*α*-induced hyperalgesia: (a) time-course curve; (b) AUCs of the time-course curve. Each point represents the mean ± SEM. Each datum represents the mean of five animals and the error bars indicate SEM. The symbols ^*∗*^ and † indicate the significance levels compared to respective controls: ^*∗∗∗∗*^*p* < 0.0001, ^*∗∗*^*p* < 0.01, ^*∗*^*p* < 0.05 (two-way ANOVA followed by Bonferroni's post hoc test); ^†††^*p* < 0.001, ^††^*p* < 0.01, ^†^*p* < 0.05 (one-way ANOVA followed by Newman–Keuls post hoc test).

The intraplantar administration of TNF-*α* reduced paw withdrawal latencies, mimicking a state of hypernociception in the animals. Both PLE (30, 100, and 300 mg kg^−1^) and morphine (3 mg kg^−1^, i.p.) increased the paw withdrawal latencies of rats significantly. The time-course curve (Figure 5(a)) revealed that PLE (300 mg kg^−1^) gave a significant reduction in the total nociception at 1, 2, and 5 h with % maximum possible effects of −23.1, 2.5, and 31.8, respectively, compared to −56.9, −36.2, and −9.3 obtained for the negative control (saline) group within the same time. The calculated AUC (Figure 5(b)) representing the total antinociceptive effect also revealed that the extract, 30 mg kg^−1^, 100 mg kg^−1^, and 300 mg kg^−1^, significantly reduced the total nociception to -31.2 (*p* < 0.05), 17.1 (*p* < 0.001), and 10.40 (*p* < 0.01), respectively, compared to -119.7 as obtained by the saline group. Correspondingly, from the time-course graph and the calculated AUC, morphine (3 mg kg^−1^) significantly inhibited hyperalgesia compared to the vehicle-treated group with a total antinociceptive effect of 124.4 (*p* < 0.0001).

### 3.7. Effect of PLE on Interleukin-1*β-* (IL-1*β-*) Induced Hyperalgesia

**Figure 6:**
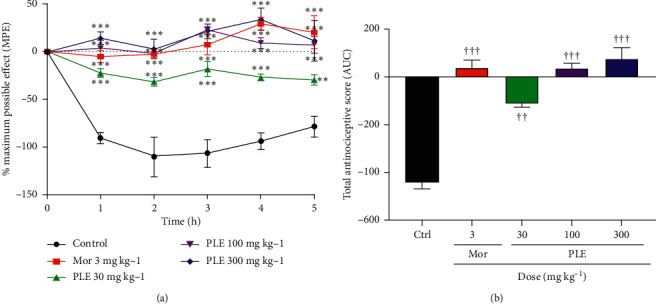
Effect of PLE and morphine on interleukin-1*β*-induced hypernociception: (a) time-course curves; (b) AUCs of the time-course curves. Data presented as mean ± SEM. The symbols ^∗^ and † indicate the significance levels compared to respective controls: ^*∗∗∗∗*^*p* < 0.001, ^*∗∗*^*p* < 0.01, ^*∗*^*p* < 0.05 (two-way ANOVA followed by Bonferroni's post hoc test); ^†††^*p* < 0.001, ^††^*p* < 0.01 (one-way ANOVA followed by Newman–Keuls post hoc test).

The results presented in Figures 6(a) and 6(b) show that IL-1*β* prominently decreased rats' paw withdrawal thresholds. PLE (30, 100, and 300 mg kg^−1^) similar to morphine (3 mg kg^−1^, i.p.) significantly reversed the hypernociception induced by IL-1*β* compared to the negative control. The time-course curve (Figure 6(a)) revealed that morphine and all doses of PLE produced a significant (*p* < 0.001) reduction in the total nociception from the 1^st^ hour to the 5^th^ hour compared to the negative control group. The calculated AUC (Figure 6(b)) also revealed that the extract at the doses of 100 mg kg^−1^ and 300 mg kg^−1^ significantly reduced the total hypernociception by producing mean total antinociceptive scores of 38.6 and 79.4, respectively, with the latter comparing better than the 40.3 obtained for morphine 3 mg kg^−1^.

### 3.8. Effect of PLE on Bradykinin-Induced Hyperalgesia

**Figure 7:**
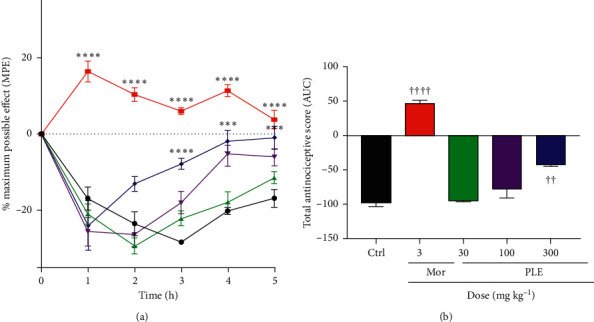
The effect of PLE and morphine on bradykinin-induced mechanical hyperalgesia in rats: (a) time-course curves; (b) AUCs of time-course curves. Data are presented as mean ± SEM. The symbols ^∗^ and † indicate the significance levels compared to respective controls: ^*∗∗∗∗*^*p* < 0.0001, ^*∗∗*^*p* < 0.01 (two-way ANOVA followed by Bonferroni's post hoc test); ^††††^*p* < 0.0001, ^††^*p* < 0.01 (one-way ANOVA followed by Newman–Keuls post hoc test).

All the animals injected with bradykinin developed hyperalgesia with a total antinociceptive score (AUC) of −97.4. From the graphs (Figures 7(a) and 7(b)), there was a significant reduction in the total nociceptive score by the highest dose of PLE (300 mg kg^−1^), which produced a total antinociceptive score of −42.6 (*p* < 0.01). This was less than the effect produced by the 3 mg kg^−1^ morphine (47.1, *p* < 0.0001). The time-course curve (Figure 7(a)) revealed that during the 3^rd^, 4^th^, and 5^th^ hour after bradykinin injection, the PLE (300 mg kg^−1^) produced a significance at (*p* < 0.0001) with a percentage of maximum possible effect of −7.8, −1.9, and −0.9, respectively, compared to that of −28.3, −20.1, and −16.8 obtained for the negative control (saline) group at the same periods. Also, during the 4^th^ hour, the extract at 100 mg kg^−1^ reduced total nociception at *p* < 0.01 with a % MPE of −5.1.

## 4. Discussion

*P. lanigera* is a promising but less explored medicinal species of the genus *Persicaria*. In this study, an HPLC fingerprint was developed to provide a reference for the correct identification and authentication of the extract from the leaves of *P. lanigera*. From the HPLC chromatogram, seven prominent peaks with their retention times, peak height, and peak areas were observed, which will serve as a fingerprint for subsequent identification of the hydroethanolic extract of *P. lanigera* leaves. The retention times for the observed peaks suggest that PLE contains constituents that are generally polar to nonpolar in nature. The fingerprint developed will also be essential for assessing the stability of the PLE in further studies as a quality control tool [18].

Hydroethanolic leaf extract of *Persicaria lanigera* (PLE) showed remarkable antinociceptive activity in the acetic acid nociception model. Although nonspecific, the acetic acid writhing test is typically used to assess the peripheral analgesic effects of drugs through intraperitoneal acetic acid injection, resulting in peritoneal cavity discomfort [19]. Pain sensation in the acetic acid-induced nociception model has been ascribed to inflammation due to the localized production and release of cyclooxygenase (COX) products such as prostaglandins (PGE_2_ and PGF_2*α*_) and lipoxygenase products [20]. Acetic acid administration may cause nociception through the activities of localized release bradykinin, IL-1*β*, IL-8, and TNF-*α* from mast cells and macrophages [21]. PLE may have induced an antinociceptive effect by inhibiting the synthesis of inflammatory or endogenous pain mediators such as bradykinin, IL-1*β*, IL-8, TNF-*α*, cyclooxygenases, COX-2, and prostaglandins produced in the peritoneal cavity. Morphine, which is a centrally acting analgesic [22], also showed a significant antinociceptive effect when used as a standard positive control, thus suggesting that the PLE could also mediate antinociception centrally like morphine, although its peripheral effect cannot be ruled out.

In the formalin test, the PLE exhibited a significant antinociceptive effect by reducing formalin-induced paw licking in both phases, although it was more potent in the second phase. Nociception induced by formalin occurs in two phases. The first phase (0–5 min) is characterized by neurogenic pain due to direct nociceptor stimulation by chemicals such as substance P, histamine, serotonin, and bradykinin [23]. The second phase (15–30 min) is signified by inflammatory pain resulting from excitatory amino acid mediators, neuropeptides, IL-33, IL-6, IL-1*β*, IL-8, PGE_2_, and kinins [24]. Our results show that PLE can inhibit both neurogenic and inflammatory pain. Since the activity of PLE was observed in both phases, it might be due to the ability of the PLE to inhibit neurogenic pain arising from histamine, serotonin, substance P, and bradykinin. Moreover, the extract pronounced inhibitory activity in the second phase, which suggests that it may have blocked the synthesis of inflammatory pain mediators such as neuropeptides, interleukins, and kinins just as revealed in the acetic acid model.

The effect of the hydroethanolic leaf extract of *Persicaria lanigera* on mechanical hyperalgesia was assessed in the Randall–Sellito test. The production of IL-1*β* post-TNF-*α* antigen administration results in the production of prostaglandin E_2,_ which ultimately results in hypernociception [5]. Also, intradermal administration of cytokines such as TNF-*α* and interleukin IL-1*β* produces significant mechanical hypernociception [25]. For this reason, anti-TNF*α* agents and interleukin-1 monoclonal antibodies are being developed as analgesics [26]. In this model, PLE (30, 100, and 300 mg kg^−1^) significantly reversed hypernociception induced by both TNF-*α* and IL-1*β*. This indicates a possible negative modulation of cyclooxygenases, hence confirming its inhibitory activity against inflammatory pain mediators as already exhibited in the acetic acid and formalin models above. Specifically, these results also show that PLE antagonizes TNF-*α*- and IL-1*β*-induced hyperalgesia.

In neurogenic inflammation, bradykinin, a potent inflammatory peptide messenger released from damaged tissues, is known to induce central sensitization in the CNS [27]. Also, an increased level of bradykinin causes the excitation of primary afferent nociceptors entering the dorsal horn of the central nervous system [28]. Moreover, bradykinin is also known as an important peripheral mediator of pain, which elicits nociception or hyperalgesia by direct stimulation of the nociceptors A*δ* and C-fibres. Bradykinin stimulates the synthesis and release of prostaglandins, nitric oxide, and neurokinins, which are second messengers, hence reducing the pain threshold [29]. Bradykinin-induced nociception works by indirectly activating PKA and directly activating B_2_ receptor-mediated phospholipase C (PLC), which results in PKC production culminating in sensitization of sensory ion channels causing hypernociception [4, 30]. The hypernociception induced by bradykinin, a marked sensitizer of the nociceptor, was reversed significantly in a dose-dependent fashion by PLE. Thus, it is possible that PLE inhibited hypernociception induced by bradykinin by either blocking the B2 receptor or PKA activation or through alternative mechanisms, but this is yet to be established.

The antinociceptive effects of PLE were also investigated in the glutaminergic system using a glutamate-induced paw licking test. Glutamate has been established to be a major excitatory amino acid neurotransmitter that functions in transmitting nociceptive signals and activates glutamate receptors. These receptors are normally implicated in nociceptive primary afferent transmissions and the development and maintenance of nociception [31]. The nociceptive behaviour evoked by glutamate occurs mainly in the peripheral, spinal, and supraspinal sites by interacting with N-methyl-D-aspartate (NMDA) and non-NMDA receptors [32]. Glutamate also induces the release of some inflammatory mediators such as nitric oxide (NO), prostaglandins, bradykinin, and NO-related substances [28]. The antinociceptive activity of PLE in this could potentially be through interference with the pain perception effects of glutamate at the periphery, spinal, or supraspinal site by inhibiting the action of the inflammatory mediators released by the glutamate. It is evident that PLE, at the doses tested and from the results, exhibited better antinociception than ketamine and, in some instances, morphine. This is exciting as it gives more credence to further development of the extract and its constituent as potent analgesics. It also an important confirmation of its traditional use to manage painful conditions.

## 5. Conclusion

The hydroethanolic leaf extract of *Persicaria lanigera* (PLE) inhibits acetic acid-induced, formalin-induced, and glutamate-induced nociception and hypernociception induced by TNF-*α*, IL-1*β*, and bradykinin. The efficacy of PLE suggests the presence of active phytochemical constituents with analgesic properties. The result obtained, therefore, confirms or verifies the folkloric use of *Persicaria lanigera* leaves in traditional medicine for managing pain-evoking disease conditions in Ghanaian communities.

## Figures and Tables

**Table 1:** Peaks representing various constituents in PLE.

Peak no.	RT (min)	Area	Height
1	2.840	27,901.2	9,375.3
2	2.893	18,283.6	8,677.5
3	3.030	188,470.8	48,391.2
4	16.441	1,852,589.7	189,965.4
5	17.445	47,533.4	6,364.3
6	17.674	292,645.2	28,301.8
7	19.043	74,733.9	10,734.7
Total		2,502,157.8	

## Data Availability

The data used to support the findings of this study may be requested and obtained from the corresponding author.
